# Prevalence of Depression among Migrants: A Systematic Review and Meta-Analysis

**DOI:** 10.3390/ijerph15091986

**Published:** 2018-09-12

**Authors:** Shea Q. Foo, Wilson W. Tam, Cyrus S. Ho, Bach X. Tran, Long H. Nguyen, Roger S. McIntyre, Roger C. Ho

**Affiliations:** 1 Department of Psychological Medicine, Yong Loo Lin School of Medicine, National University of Singapore, Singapore 119228, Singapore; pcmrhcm@nus.edu.sg; 2 Alice Lee Centre for Nursing Studies, Yong Loo Lin School of Medicine, National University of Singapore, Singapore 119077, Singapore; nurtwsw@nus.edu.sg; 3 Department of Psychological Medicine, National University Health System, Singapore 119228, Singapore; su_hui_ho@nuhs.edu.sg; 4 Institute for Preventive Medicine and Public Health, Hanoi Medical University, Hanoi 100000, Vietnam; bach@jhu.edu; 5 Institute for Global Health Innovations, Duy Tan University, Da Nang 550000, Vietnam; long.ighi@gmail.com; 6 Mood Disorders Psychopharmacology Unit, University Health Network, University of Toronto, Toronto, ON M5T 1R8, Canada; roger.mcintyre@uhn.ca

**Keywords:** depression, migrant, migration

## Abstract

As the number of migrants worldwide increases, it is worthwhile to examine the extent to which depression has affected this group of often vulnerable individuals. The purpose of this systematic review and meta-analysis is to examine the aggregate prevalence of depression among international migrants and to explore the variations in prevalence with demographic and educational factors. A search was conducted on the online databases PubMed and ScienceDirect whole using the terms “depression”, “depressive disorder”, “immigration”, “immigrant”, “migration”, and “migrant”. A total of 25 studies met our inclusion criteria. A random-effects model meta-analysis calculated an aggregate prevalence of 15.6% among migrants. Heterogeneity was identified by meta-regression and subgroup analyses, and the level of educational attainment, employment status, and length of residency spent in country of migration were found to be significant moderators contributing to depression prevalence. In conclusion, newly arrived migrants appear to be susceptible to developing depression and it is imperative that more in the form of preventive strategies and increased assistance be incorporated to ensure their psychological wellbeing and improve their mental health outcomes. Further research should be conducted to better understand the risk of psychiatric disorders among members of this subpopulation.

## 1. Introduction

Globalization in the 21st century has translated into an increasingly interconnected world, with more people than ever before in human history moving across borders in search of better employment opportunities and lifestyles. Accordingly, a steep upward trend in international migrant patterns has been observed in recent years, with an estimated 258 million migrants worldwide as of 2017, which is up from 220 million in 2010 and 173 million in 2000 [1]. For many migrants, uprooting to a new country is often accompanied by drastic changes in multiple aspects of their lives. The process of assimilating to new surroundings and cultural practices can lead to considerable levels of acculturative stress [2,3], which in turn has been linked with the manifestation of psychiatric disorders (e.g., depression) [4,5]. Globally, depression has been identified as the leading cause of illness and disability, with an aggregate point, one-year and lifetime prevalence of 12.9%, 7.2%, and 10.8%, respectively [6]. In its most severe form, it can lead to suicide. Approximately 800,000 people die from suicide annually, and it remains the second leading cause of death in 15 to 29 year-olds [7].

With that being said however, evidence of a definite cause-and-effect relationship between migration, acculturative stress and subsequent development of depression appears arbitrary at best. The association between migration and depression remains contentious and numerous studies have found the link to be inconsistent [8,9]. Given the conflicting evidence available with current empirical research, the relationship between migration and depression presents worthwhile questions to examine. The aim of this systematic review and meta-analysis is therefore to explore the aggregate prevalence of depression among international migrants, and to investigate for any possible variations in prevalence with demographic and educational factors.

## 2. Materials and Methods

Given the heterogeneity among international migrants, multiple varying definitions of the term “migrant” exist in literature, but none has so far been universally accepted. For the purposes of this paper, we adopted the definition as provided by the United Nations Convention of the Rights of Migrants according to UNESCO (The United Nations Educational, Scientific and Cultural Organization) [10], which defines “migrant” as a term “covering all cases where the decision to migrate is taken freely by the individual concerned, for reasons of ‘personal convenience’ and without intervention of an external compelling factor”. Children, refugees, asylum seekers as well as second generation and later migrants were therefore excluded from this definition.

### 2.1. Search Strategy

A search strategy was conducted using the online databases PubMed and Science Direct from March 2018 to April 2018. Keywords used included the terms “depression” OR “depressive disorder” OR “major depressive disorder” AND “immigra*” OR “migration” or “migrant”. No limitations were imposed with regard to date of publication so as to maximize the retrieval of relevant articles.

### 2.2. Inclusion and Exclusion Criteria

Studies were included in this systematic review and meta-analysis only if they were quantitative cross-sectional or longitudinal studies by design. Only those that had analysed the prevalence of depression among migrants as part of their study or had provided sufficient relevant data to calculate said prevalence, and had done so while using validated standardised instruments were considered. The studies also had to involve more than 50 migrants as part of their pool of participants and must have been published in peer-reviewed journals with an English abstract and full-text accessible for full review. Studies were excluded if they were interventional studies, cohort studies, case-control studies, case reports, case series, newspaper articles, magazine articles, conference papers, or commentaries. Those that had abstracts or full-texts in languages apart from English, or did not provide sufficient relevant data for the prevalence of depression among migrants to be calculated were also excluded. To ensure comparability across diagnostic measurements, only adult populations were included and this excluded studies that had involved participants aged either less than 16 years or more than 65 years at the beginning of the study. In accordance with the aforementioned definition of “migrant”, studies that had involved refugees, asylum seekers, rural-to-urban internal migrants, and second generation or later generation migrants were excluded, unless they had defined the different groups clearly and had analyzed data for each group independently such that the specific aggregate prevalence of depression in solely first generation international migrants could be retrieved.

### 2.3. Study Selection and Data Extraction

Study selection was done based on the Preferred Reporting Items for Systematic Reviews and Meta-Analyses (PRISMA) Flow Diagram [11]. Results from the electronic online database search were first downloaded into EndNote X8.2 (Clarivate Analytics, Philadelphia, PA, USA) in order for duplicate results to be removed both electronically and manually. The titles and abstracts of the remaining articles were then individually screened for relevance. The inclusion and exclusion criteria were then applied to remove the ineligible articles. Following which, full-texts of eligible articles were retrieved and subjected to a full review. Data that were extracted from the final pool of eligible articles were then documented on an Excel spreadsheet to include the following information: first author’s last name; year of publication; country of migration; country of emigration; study design; methods utilized to assess prevalence of depression and cut-off scores used therein; number of migrants (and natives, if applicable); mean age of migrants (and natives, if applicable); percentage of male migrants (and natives, if applicable); percentage of migrants currently employed (and natives, if applicable); percentage of migrants who received high school or college education (and natives, if applicable); percentage of migrants who indicated their marital status as single (and natives, if applicable); length of residency of migrants; and, prevalence of depression among migrants (and natives, if applicable).

### 2.4. Statistical Analyses

Statistical analyses were conducted while using the Comprehensive Meta-Analysis Version 2.0 programme (Biostat, Englewood, NJ, USA). A random effects model was adopted to calculate the aggregate prevalence and 95% confidence intervals (CIs) in view of the expected heterogeneity across the studies. The random-effects model was used as it not only assumes varying effect sizes between studies, but also takes into account differing study designs and study populations [12]. Heterogeneity between studies was assessed and quantified via Cochran’s chi-square and *I*^2^ statistic, respectively. The *I*^2^ statistic describes the proportion of variation across the studies that is due to heterogeneity rather than chance [13,14]. As a guide, *I*^2^ values of 25% may be considered low, 50% moderate and 75% high [15]. To identify the different moderators that might have contributed to the heterogeneity or the observed variations in aggregate depression prevalence between studies, a mixed-effects meta-regression was completed [16]. The covariates examined were mean age of migrants, percentage of male migrants, percentage of single migrants, percentage of migrants with high school education and college education, percentage of migrants currently employed, and length of residency spent in the country of migration (in years). The regression coefficients, associated *z* values and *p* values were reported in the meta-regression analysis. Publication bias was evaluated using funnel plots and the modified Egger’s linear regression test [17]. Significance was set at *p* < 0.05.

## 3. Results

**Figure 1:**
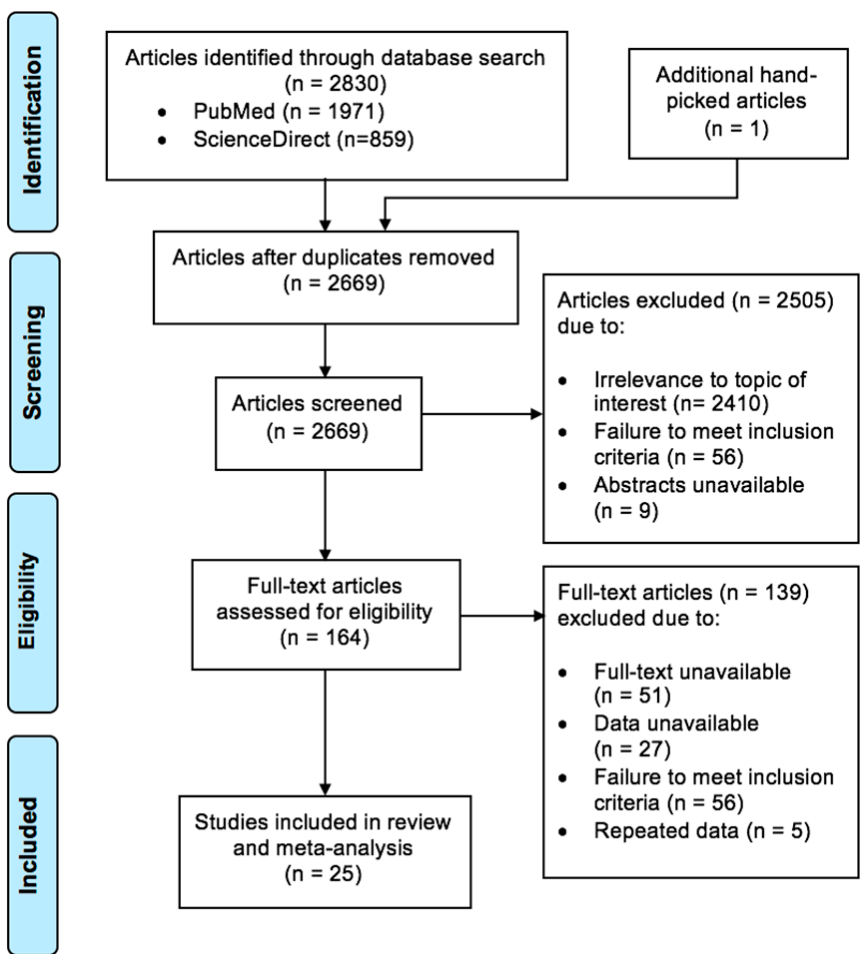
Process of systematic selection using the Preferred Reporting Items for Systematic Reviews and Meta-Analyses (PRISMA) flow chart.

Of the 2831 results that were obtained from the initial online electronic search, a total of 25 studies were finally included in this review. The process of study selection is summarised with the PRISMA flow diagram, as depicted in Figure 1. All the final included studies were cross-sectional in design and had utilized validated standardised instruments as methods to assess depression prevalence. The various methods that were used in the final included studies were as follows: the Beck Depression Inventory (BDI) [18], the Beck Depression Inventory 2nd edition (BDI-II) [19], the Centre for Epidemiologic Studies Depression Scale (CES-D) [20] and its Korean variation (CES-D-K) [21], the World Health Organization Composite International Diagnostic Interview (CIDI) [22] and its Turkish computer-assisted version (CIDI DIA-X) [23], the Disaster-Related Psychological Screening Test (DRPST) [24], the Hopkins Symptom Checklist-25 (HSCL-25) [25], the Mini International Neuropsychiatric Interview (MINI) [26], the Patient Health Questionnaire-9 (PHQ-9) [27] and its two-item version (PHQ-2) [28], and the Primary Care Evaluation of Mental Disorders (PRIME-MD) [29]. There were a total number of 31,391 participants included in this review, with 16,121 migrants and 15,270 native respondents covered altogether. The prevalence of depression and characteristics of each included article are presented in Appendix A.

### 3.1. Demographic Data of Participants

The mean age of migrants ranged from 25.96 to 47.3 years. Among the 25 included studies, 16 had assessed depression prevalence solely among migrants, whereas the other nine had provided data for both migrants and native participants. In these nine studies, the mean age of native participants ranged from 28.7 to 39.0 years.

The countries in which the studies were conducted were divided into two large categories: the United States of America and the rest of the world. This was decided in view of the fact that 11 out of the 25 final included studies had been conducted in the former group. The latter group in turn comprised of one study from Australia, two from Canada, one from the Dominican Republic, one from Greece, one from Hong Kong, one from New Zealand, one from South Korea, two from Spain, and two from Taiwan. For feasibility of subgroup analysis, these studies were pooled together as a single subgroup since there were inadequate studies from each continent. The 25 final included studies were also categorized based on the vernacular language of the country in which they had been conducted into English speaking and non-English speaking countries. Of these 25 studies, 15 were from countries that were English speaking and 10 were from countries that were non-English speaking.

### 3.2. Aggregate Prevalence of Depression

**Figure 2:**
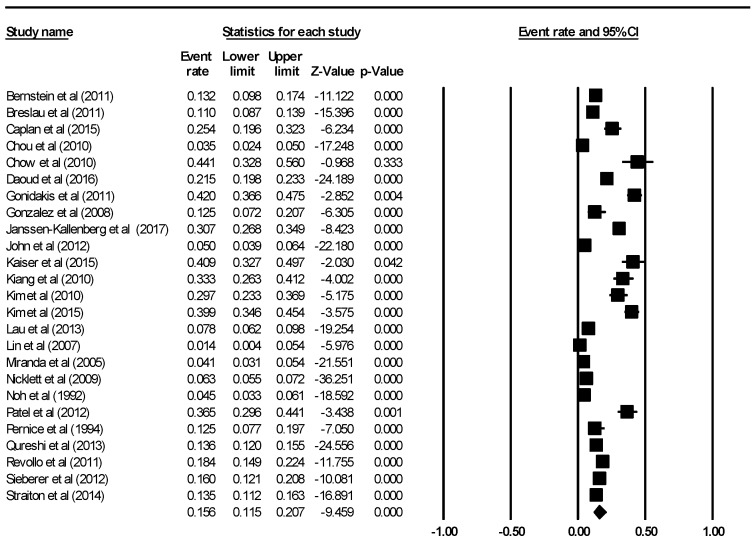
Forest plot of aggregate prevalence of depression among migrants using data from all 25 included studies.

The aggregate prevalence of depression among migrants based on all 25 final included studies while using the random-effects model was 15.6% (95% CI: 11.5–20.7%, Q value = 1191.213, *df* = 24, tau^2^ = 0.760). This is demonstrated using the forest plot as depicted in Figure 2. There was a significant high level of heterogeneity across the included studies (*I*^2^ = 97.985, *p* < 0.001).

### 3.3. Meta-Regression and Publication Bias

Results from the meta-regression analysis can be found in Table 1. The percentage of migrants with high school education (*B* = 0.022, *z* = 8.357, *p* = 0.000) and college education (*B* = 0.008, *z* = 4.325, *p* = 0.000), percentage of migrants currently employed (*B* = −0.0138, *z* = −8.482, *p* = 0.000) and length of residency spent in country of migration (*B* = −0.088, *z* = −11.128, *p* = 0.000) were identified as significant moderators that contributed to heterogeneity between the studies. However, the percentage of male migrants (*B* = −0.001, *z* = −0.385, *p* = 0.700), percentage of single migrants (*B* = −0.001, *z* = −0.850, *p* = 0.395), and mean age of migrants (*B* = −0.001, *z* = −0.309, *p* = 0.757) were non-significant moderators. There was no evidence of publication bias (intercept = −0.71, 95% CI: −7.582–6.174, t = 0.214, *df* = 23, *p* = 0.832).

### 3.4. Subgroup Analysis

Results from the subgroup analysis can be found in Table 2. Among the 25 final included studies, 17 had provided relevant data on mean age of migrants. Between the different age groups, the greatest depression prevalence was reported in the youngest group (25–34 years) at 14.8% (95% CI: 5.5–33.8%). This was followed by the second-youngest age group (35–44 years) at 12.8% (95% CI: 5.8–26%). The oldest age group (45 years and above) had the lowest prevalence of depression at 11.8% (95% CI: 6.6–20.1%). The differences were not significant (*p =* 0.616). Stratification according to country of immigration was performed in all 25 final included studies. Studies conducted in the United States of America reported lower depression prevalence at 14.8% (95% CI: 8.4–24.8%), while those that were conducted across the rest of the world reported higher depression prevalence at 16.8% (95% CI: 10.1–26.6%). The differences were not significant (*p* = 0.733). Comparison was made also with regard to the year of publication of included studies. The depression prevalence for studies published in 2010 or earlier was 11.0% (95% CI: 4.9–23.1%) and for studies published after 2010 was 19.6% (95% CI: 14.1–26.6%). The difference was not significant (*p* = 0.130). Subgroup analysis according to vernacular language showed that studies conducted in English speaking countries had a lower depression prevalence at 13.8% (95% CI: 8.8–21.0%) when compared to those conducted in non-English speaking countries at 19.3% (95% CI: 10.0–33.8%) but the difference was not statistically significant (*p* = 0.360). A combined odds ratio was calculated for the nine studies that had provided relevant data comparing depression prevalence between migrants and native participants. The data showed that in comparison with native participants, migrants had relatively lower odds of depression (OR: 0.865, 95% CI: 0.549–1.362), but this difference was however not significant (*p* = 0.530).

## 4. Discussion

Following analysis of data obtained from 16,121 migrant participants across 20 different countries, this systematic review and meta-analysis reports an aggregate prevalence of depression among international migrants of 15.6%.

Although such a figure suggests that depression is a common and substantial mental health problem affecting migrants worldwide, we did not note any significant difference in depression prevalence between migrants and native participants. This appears to correspond with the findings of a previous systematic review that found no conclusive evidence of an increase in risk of depression or other mood disorders in general among members of the migrant community [30]. It is difficult to account for such a finding, as one would expect the acculturative stress of migration to be an important risk factor for depression. The same paper however attributed this apparent disparity to a selection hypothesis, which postulates that those genetically predisposed to mood disorders develop stronger attachments to people in their home countries and are therefore less likely to migrate [31]. An alternative explanation ties in the concept of resilience, a construct that represents positive adaptation, social, and psychological competence in times of significant adversity or trauma [32]. It is possible that migrants who voluntarily uproot themselves may possess resiliency factors not shared in common with natives, which explains why no differential risk for adverse mental outcomes were noted between the two groups. Migration may also at times be associated with upward social mobility and improved quality of life for individuals who move away from environmental pathogens such as high crime and unemployment rates. The improved psychosocial outcomes from this change in environment may negate the increased risk of depression from acculturative stress. Therefore, to categorically conclude that the process of migration has minimal association with depression without first accounting for factors specific to migrant individuals and their country of migration would undoubtedly be overt reductionism.

The wide range of populations that were examined in this systematic review and meta-analysis contributed to the significant heterogeneity in depression prevalence across studies. Meta-regression analysis identified moderators, including level of educational attainment, employment status, and length of residency spent in country of migration as significant contributors to the heterogeneity in aggregate prevalence. Studies which included a larger percentage of migrants who had graduated from high school and college tended to report greater depression prevalence. Given that education has long been regarded as a vehicle for social mobility, such a finding would appear to be contrary to what should be expected. However, various possible explanations for this observed trend have been proposed. An earlier study found that the prevalence of mood dysfunction was greater in Asian immigrants with higher education who had immigrated at a younger age when compared to those with lower educational attainment but who had immigrated at older ages [33]. This implies that the association between the level of educational attainment and depression may in fact be confounded by specific factors related to the migration process itself such as an individual’s age at the time of migration. Unfortunately, we could not investigate these factors in this paper since only a limited number of studies had provided the relevant data. Further research is therefore necessary and warranted for any conclusions to be drawn.

Apart from the confounding effect of other moderators, a separate study also found that educational level does not always directly translate to equivalent economic opportunity in ethnic minorities [34], of which migrants often find themselves a part of upon uprooting to a foreign land. Highly educated migrants may experience employment difficulties due to language difficulties or potential employers’ devaluation of foreign education credentials [35]. It is also possible that a higher level of educational attainment may increase awareness of social injustices and racial discrimination, with such feelings of perceived unfairness in turn resulting in feelings of distress that eventually manifest as mood dysfunction [36].

The somewhat paradoxical relationship between level of educational attainment and depression may also be accounted for by increased levels of distress caused by a discrepancy between one’s lifestyle and economic status [37]. Many a times, increased level of educational attainment may heighten expectations for a certain standard of living. Failure to realise such expectations may result in stress and ultimately depression.

Employment status was found to be a significant moderator for depression prevalence among migrants, with current employment associated with lower depression prevalence. This comes as no surprise as the association between unemployment and poor mental health has long been established [38,39]. A previous meta-analysis found evidence to support a causal relationship between the two, as substantiated by an individual’s mental well-being typically declining following unemployment, but improving after subsequent reemployment [40]. A number of theories have been proposed to explain such an association.

One such theory postulated that mental distress arising from unemployment stems primarily from the lack of five positive latent functions of employment [41]. It posits that employment in fact imposes a time structure on the day, increases social contact with others, offers one a sense of collective purpose, allows for increased status, and encourages activity. Unemployment inevitably causes the deprivation of these important latent functions, in turn resulting in mental distress and poor quality of life [42]. Such an effect is all the more pertinent among migrants, who are often blighted with difficulties involving social and economic integration in their newly adopted country. This is corroborated by previous research that reported that the negative effects of unemployment are likely increased among minority groups [43]. It is possible that the social and financial instability unemployment confers upon individuals aggravates the acculturative stress socioeconomically disadvantaged migrants already face and increase their susceptibility to mood dysfunction.

An inverse relationship between depression prevalence and length of residency was also observed. Studies that had included migrants with a shorter duration of stay in their country of migration were inclined to report greater depression prevalence. This is largely consistent with reports in the literature [44] and may be attributed to the fact that levels of acculturative stress typically peak in the early post-migration phase. During this period of assimilation, migrants commonly encounter problems with integration as they adjust to a host of changes in their lives, including chaotic disruptions to their social networks and challenges to their cultural values. The high levels of acculturative stress that accompany this process of adaptation may in turn result in increased mental distress and subsequent mood dysfunction.

## 5. Strengths and Limitations

The strengths of this review include an extensive literature search in identifying a large number of relevant articles, established methodology using the PRISMA guidelines, and a comprehensive meta-analysis that included regression and subgroup analysis. Studies that were included were also conducted in a broad range of diverse countries, which meant that any differential risk as a result of cultural or ethnic differences was minimized. Other strengths include a lack of significant publication bias and the application of a random-effects model in establishing robust aggregate depression prevalence among migrants.

Nonetheless, several limitations have been identified. First, this review has a high level of heterogeneity typical of meta-analyses of large number of studies. Second, meta-regression analyses were only performed on selected demographic moderators as there was limited data available on other moderators including age of migration. Data pertaining to pre-migration characteristics such as socio-economic background and psychological health status was also inadequate. Third, meta-regression merely denotes an observational association and is limited by ecological fallacy [45]. Fourth, all of the included studies were cross-sectional by design, which meant that a temporal causality between migration and depression could not be identified. Further reviews in the future should include more longitudinal studies to address this. Lastly, our inclusion and exclusion criteria meant that studies that were published in languages apart from English were excluded from our analysis. Studies were also retrieved primarily from two online databases, and thus this review may not be entirely representative.

## 6. Conclusions

In conclusion, this systematic review calculated an aggregate prevalence of depression among migrants of 15.6%. Meta-regression analyses showed that level of educational attainment, employment status, and length of residency were significant contributors to the high level of heterogeneity in prevalence of depression.

Our findings highlight the fact that migrants, particularly those newly arrived and unemployed, remain susceptible to developing mood dysfunction. With the number of migrants worldwide set to increase for the foreseeable future, such a trend is likely to persist unabated. These findings have underlined the gap in existing social and cultural supports for recent migrants, and it is imperative that more community resources be made available to help ease the process of social and economic integration. Screening for mood dysfunction should also be commenced early among the members of this vulnerable population.

Future research should attempt to elucidate the impact pre-migration mental health and host country factors has on depression prevalence among migrants. Qualitative studies that assess the role that specific cultural factors has on depression predisposition should also be pursued.

## Figures and Tables

**Table 1:** Results for meta-regression analysis.

Predictor	No. of Studies Used	Univariate Coefficient	*z* Value	*p* Value	Estimated tau^2^
Percentage of migrants with college education	16	0.00828	4.32484	0.00002	0.72087
Percentage of migrants with high school education	14	0.02189	8.35707	0.00000	0.80752
Percentage of migrants currently employed	14	−0.01378	−8.48239	0.00000	0.67017
Length of residency (years)	12	−0.08804	−11.12773	0.00000	1.15403
Percentage of male migrants	23	−0.00054	−0.38529	0.70002	0.81583
Percentage of single migrants	18	−0.00139	−0.84998	0.39533	0.83410
Mean age of migrants	17	−0.00132	−0.30879	0.75748	0.91546

**Table 2:** Results for subgroup analysis.

Category	Subgroup	No. of Studies	Pooled Prevalence, %	95% CI	*p* Value in Between-Group Comparison
Mean age (years) of migrants	25–34	8	14.8	5.5–33.8	0.616
	35–44	5	12.8	5.8–26.0	
	45 and above	4	11.8	6.6–20.1	
	Overall	17			
Country of immigration	United States	11	14.8	8.4–24.8	0.733
	Rest of the world	14	16.8	10.1–26.6	
	Overall	25			
Vernacular language	English speaking	15	13.8	8.8–21.0	0.360
	Non-English speaking	10	19.3	10.0–33.8	
	Overall	25			
Year of Publication	2010 or earlier	10	11.0	4.9–23.1	0.130
	Later than 2010	15	19.6	14.1–26.6	
	Overall	25			

## Appendix A

**Table A1:** Descriptive data of the 25 included studies for review.

Authors, Year	Country of Migration	Country of Emigration	Study Design	No. of Participants (Migrants /Natives)	Age of Migrants, Mean Years	Male Migrants, %	Migrants Currently Employed, %	Level of Education of Migrants (High School/College), %	Marital Status of Migrants (Single), %	Length of Residency (Years)	Method to Assess Depression/Cut-Off Score	Prevalence of Depression in Migrants, %
Bernstein et al. (2011) [46]	United States	South Korea	Cross-sectional	304/0	46.7	43.4	NR	29/53.3	34.9	14.8	CES-D-K; Cut-off = 21	13.2
Breslau et al. (2011) [47]	United States	Mexico	Cross-sectional	554/0	NR	46.751	NR	NR	NR	NR	WHO WMH-CIDI; Cut-off = NR	11
Caplan et al. (2015) [48]	United States	Dominican Republic, Ecuador, Colombia and other Latino countries	Cross-sectional	177/0	NR	27.119	69	31.073/11.299	NR	NR	PHQ-9; Cut-off = 10	25
Chou et al. (2010) [49]	Taiwan	Vietnam, Indonesia, China and others	Cross-sectional	801/801	31.05	0	36.330	27.840/5.493	0	6.58	DRPST; Cut-off = 2	3.5
Chow et al. (2010) [50]	Hong Kong	China	Cross-sectional	68/0	NR	0	14.706	79.412/11.765	13.235	NR	CES-D; Cut-off = 16	45
Daoud et al. (2016) [51]	Canada	Varied countries	Cross-sectional	2066/0	47.3	45.257	60.683	NR/51.019	36.641	NR	CES-D; Cut-off = 16	21.8
Gonidakis et al. (2011) [52]	Greece	Albania, Nigeria, Iraq, Pakistan and other countries	Cross-sectional	317/0	31.6	53.628	28.391	NR	42.271	3.417	CES-D; Cut-off = 16	42
Gonzalez et al. (2008) [53]	United States	Mexico	Cross-sectional	96/57	NR	41.053	NR	46.875/6.250	71.875	11.5	BDI-II; Cut-off = 20	12.5
Janssen-Kallenberg et al. (2007) [54]	Germany	Turkey	Cross-sectional	502/151	NR	NR	NR	NR	NR	NR	CIDI DIA-X Ver. 2.8; Cut-off = NR	30.7
John et al. (2012) [55]	United States	China, Vietnam, Philippines and other Asian countries	Cross-sectional	1193/335	40	52	91	NR	26	15.8	WHO WMH-CIDI; Cut-off = NR	5
Kaiser et al. (2015) [56]	Dominican Republic	Haiti	Cross-sectional	127/0	33.4	58.268	NR	NR	81.890	NR	BDI; Cut-off = NR	40.9
Kiang et al. (2010) [57]	United States	Mexico	Cross-sectional	150/0	29.67	54.7	71.3	66.6/8.0	32.4	1.93	CES-D; Cut-off = 16	62.667
Kim et al. (2010) [58]	United States	South Korea	Cross-sectional	172/0	40.9	30.814	NR	NR	3.489	13.06	CES-D; Cut-off = 16	29.9
Kim et al. (2015) [59]	South Korea	Vietnam, China, Philippines and other Asian countries	Cross-sectional	316/0	NR	0	NR	39.103/27.244	0	NR	CES-D; Cut-off = 16	39.9
Lau et al. (2013) [60]	United States	China, Vietnam, Philippines and other Asian countries	Cross-sectional	845/185	43.42	0	NR	16.92/18.03	NR	NR	WHO WMH-CIDI; Cut-off = NR	7.8
Lin et al. (2007) [61]	Taiwan	Vietnam	Cross-sectional	143/0	25.96	0	NR	NR	0	4.02	BDI-II; Cut-off = 14	1.4
Miranda et al. (2005) [62]	United States	African and Caribbean countries	Cross-sectional	1186/7965	32.1	0	30.860	34.235/26.289	40.894	NR	Prime-MD; Cut-off = NR	4.1
Nicklett et al. (2009) [63]	United States	Asian and Latino countries	Cross-sectional	3056/0	39.84	46.171	NR	NR	NR	NR	WHO WMH-CIDI; Cut-off = NR	6.4
Noh et al. (1992) [64]	Canada	South Korea	Cross-sectional	860/0	45	53	74.4	52.5/39.3	17.4	12	CES-D; Cut-off = 16	4.5
Patel et al. (2012) [65]	United States	Bangladesh	Cross-sectional	167/0	35	0	18.563	41.317/22.754	1.198	5.6	PHQ-2; Cut-off = 3	36.5
Pernice et al. (1994) [66]	New Zealand	Britain and Pacific islands	Cross-sectional	120/0	NR	NR	NR	NR	NR	5	HSCL-25; Cut-off = 1.75	12.4
Qureshi et al. (2013) [67]	Spain	Varied countries	Cross-sectional	1503/1503	32.5	38.656	64.118	46.206/22.633	50.101	NR	MINI; Cut-off = NR	13.6
Revollo et al. (2011) [68]	Spain	Ecuador, Bolivia, Peru, Columbia and other Latin American countries	Cross-sectional	414/0	34.05	27.536	68	56.280/28.744	45.894	NR	MINI; Cut-off = NR	18.4
Sieberer et al. (2012) [69]	Germany	Poland, Russia, Kazakhstan, Turkey and other countries	Cross-sectional	275/2378	NR	27.106	100	69.004/41.912	NR	22.5	CES-D; Cut-off = 23	16
Straiton et al. (2014) [70]	Australia	Varied countries	Cross-sectional	709/1895	46.9	53.5	75.8	NR	24.1	NR	CES-D; Cut-off = 16	13.5

BDI: Beck Depression Inventory, BDI-II: Beck Depression Inventory 2nd edition, CES-D: Centre for Epidemiologic Studies Depression Scale, CES-D-K: Centre for Epidemiologic Studies Depression Scale Korean variation, CIDI: World Health Organization Composite International Diagnostic Interview, CIDI DIA-X: Turkish computer-assisted version of the World Health Organization Composite International Diagnostic Interview, DRPST: Disaster-Related Psychological Screening Test, HSCL-25: Hopkins Symptom Checklist-25, MINI: Mini International Neuropsychiatric Interview, PHQ-9: Patient Health Questionnaire-9, PHQ-2: 2-item version of the Patient Health Questionnaire-9, PRIME-MD: Primary Care Evaluation of Mental Disorders. NR: Not Reported.
